# Mental health literacy at the public health level in low and middle income countries: An exploratory mixed methods study in Vietnam

**DOI:** 10.1371/journal.pone.0244573

**Published:** 2020-12-31

**Authors:** Hoang-Minh Dang, Trung T. Lam, Anh Dao, Bahr Weiss

**Affiliations:** 1 Center for Research, Information and Service in Psychology, VNU University of Education, Hanoi, Vietnam; 2 Da Nang Psychiatric Hospital, Danang, Vietnam; 3 Department of Psychology and Human Development, Peabody College, Vanderbilt University, Nashville, TN, United States of America; Chiang Mai University Faculty of Medicine, THAILAND

## Abstract

**Purpose:**

Mental health literacy (MHL) is key for mental health development, particularly in low-and-middle-income countries (LMIC) where mental health resources are limited. MHL development can be thought of as occurring at two levels: the *individual person level* (via direct contact, with specifically-targeted individuals), and the *public health level* (via indirect contact through public media, targeting the general public). Each approach has advantages and disadvantages.

**Methods:**

The present mixed methods study assessed the status of and best approaches for development of mental health literacy in the Southeast Asian LMIC Vietnam. Because there has been relatively little discussion of MHL development at the public health level, this assessment focused on the public health level, although not exclusively. Because mental health professionals generally have the most in-depth understanding of their mental health system, study participants were 82 Vietnamese mental health professionals who completed a quantitative survey, with 48 participating in focus groups.

**Results:**

Most of the professionals viewed MHL in Vietnam as low or very low, and that it was difficult or very difficult for the general public to find effective mental health services. Main barriers underlying these problems and more generally for developing MHL in Vietnam identified in the focus groups were: (a) misinformation in the media regarding mental health and mental illness; (b) lack of licensure for non-medical mental health professionals (e.g., psychologists; social workers); (c) lack of interest in mental health from upper-level leadership.

**Conclusions:**

To the best of our knowledge, this is the first study assessing professionals’ perceptions regarding mental health literacy at both the public health and individual-person levels. Although sampling was restricted to Vietnamese professionals, results may provide initial preliminary guidance for other LMIC considering mental health literacy development at multiple levels.

## Introduction

The term “mental health literacy” was defined by Jorm [1] as knowledge and beliefs about mental health disorders that aid in their recognition, management, and / or prevention. This definition of mental health literacy was later expanded to include several components, including (a) understanding of mental health disorders as health-related entities and (b) their general characteristics, causes, and treatments; (c) decreased stigma towards mental illness; and (d) one’s ability to promote positive mental health and effective help-seeking for mental health problems as necessary [2]. Mental health literacy has been found to be an important predictor of supportive attitudes towards mental health problems, and towards help-seeking for the self and for others [3]. Mental health literacy thus is a central component to mental health support and development.

### Two levels of mental health literacy development

Mental health literacy development can be thought of as operating at two levels. The first level is the *individual person level*, which involves development of mental health literacy through (a1) direct contact with (a2) specifically targeted persons. This includes, for instance, mental health literacy programs provided in specific schools for specific identified staff and/or students. For example, Kutcher, Wei [4] provided a three day mental health literacy training to 61 Tanzanian school teachers via their *African Guide*. Conversely, the second level at which mental health literacy development can occur is the *public health level*, which is broader and involves (b1) non-direct contact (e.g., via television and the internet) targeting (b2) the general public rather than specific individuals, via public health campaigns. The Australian “*beyondblue*” national depression program, for instance, involved a variety of community awareness campaigns (via the TV, etc.) intended to increase the general public’s mental health literacy regarding depression.

### Effective mental health help-seeking

Help-seeking behavior as part of mental health literacy extends beyond help-seeking per se. Rather, it involves the ability to find mental health services and providers that are likely to be *effective*, as not all mental health-related programs are effective. In high-income countries (HIC) such as the United States, the United Kingdom, etc., there typically are a number of factors supporting access to effective (vs. non-effective) treatments and providers [5, 6]. For instance in most HIC, government regulations require that individuals who provide “health-related” services (including “mental health”) be licensed by the state, with appropriate formal degree training, and passage of knowledge and ethics tests. Individuals who use specific professional labels (e.g., “psychiatrist”; “psychologist”; “social worker”) must have appropriate training within the stated discipline, and the appropriate degree and licensure. In HIC, insurance companies including national insurance plans generally only reimburse for services that have at least a moderate scientific basis for their effectiveness. Thus, although far from perfect, in HIC there are a number of structures that support the identification and use of services and providers likely to be effective [5, 6].

In many low- and middle-income countries, in contrast, these structures are not yet in-place or are not consistently enforced [7, 8]. In Vietnam, the target country of the present study, individuals without formal training in mental health or clinical psychology can call themselves a “psychologist”, and there is not licensure for psychologists. Similarly, individuals without formal training in mental health or clinical psychology can provide psychological treatment for mental health disorders without oversight of their competency or the treatments they provide [7]. Traditional healers and fortune tellers can publicly claim to “cure” depression [9]. It thus is more challenging and difficult for the public in LMIC such as Vietnam to find services and providers likely to be effective and competent in helping with mental health problems which, given the importance of finding mental health services likely to be effective, represents an important mental health literacy challenge.

### Mental health literacy at the public health level

Mental health literacy training can increase individuals’ effective mental health help-seeking, including in LMIC such as Vietnam. For instance, a recent study [10] conducted in 20 secondary schools in Danang, Vietnam involved 80 teachers who participated in a three-day mental health literacy teacher training, and 2,538 of their students who received a 5-week classroom-based student mental health literacy curriculum. Both teachers and students showed significant increases in mental health help-seeking skills, including the ability to identify appropriate mental health services [10]. It is important to note that this mental health literacy intervention was conducted at the *individual person level*. Relative to the *public health level*, mental health literacy training such as this at the *individual person level* has a number of advantages. First and foremost, there is extended direct contact between the individual and the trainer, where detailed information can be provided with questions and uncertainties answered, in the context of a trusting professional relationship. Such an approach thus can overcome many of the challenges inherent in finding appropriate mental health services in countries like Vietnam.

One limitation for programs such as this is, however, that the proportion of the population that they reach is small (approximately .00003 of the Vietnamese population, in the present case) and generally constrained to relatively easy to access groups (in the present case, teachers and students as opposed to, say, agricultural workers). To achieve improved population-level mental health literacy and appropriate help-seeking, mental health literacy development at the *public health level* is necessary. One potential challenge to developing mental health literacy at the public health level is the lack of accuracy of much of the publicly available mental health information. This issue of course is not restricted to LMIC but also occurs in HIC with, for instance, the anti-science movement in North America and Europe [11], and the proliferation of “super food” remedies [12] claimed to cure almost any disease or condition. But for the various reasons noted above, in LMIC the task of helping the general public find effective mental health treatments, which is at least in part the responsibility of the mental health and educational professional communities, appears significantly more challenging. Objective reviews of scientific evidence for treatments’ effectiveness can be presented online (e.g., the Cochrane Collaboration; www.Cochrane.org), but how to convince the general public regarding the value of such information is unclear: Anyone can assert a scientific basis for their claims regardless of the actual scientific evidence as with, for instance, the anti-vaccine movement in the West [13]. In HIC with significant oversight, the formal health care system provides some guidance (e.g., it will not reimburse for psychoanalysis), but in many LMIC such as Vietnam insurance reimbursement for psychological and behavioral treatments for mental health problems is in the beginning stages of development, providing no guidance [14].

### Present study

Thus, because professionals will be a center of change and provide solutions to develop mental health literacy at the public health level, understanding professionals’ perspective on these issues is a critical component to mental health literacy development. Given that a central goal of the study was to provide guidance on how mental health literacy can best be developed at the public health level, the study focused on the perceptions of mental health professionals who, as noted above, are most connected to the health care system and understand its potential to promote mental health literacy. Therefore, the specific aims this study were (a) to quantitively examine Vietnamese professionals’ perceptions of (a1) the current status of mental health literacy and mental health services in Vietnam and (a2) their evaluations of perceived barriers and possible solutions for public information seeking of effective mental health support; and (b) to qualitatively explore in more depth their perceptions of these issues. Given the value but particular challenges of mental health literacy development at the public health level, these discussions focused on the public health level.

## Materials and methods

### Study design

A mixed methods design was used that included a questionnaire survey as well as four focus groups conducted in Hanoi, Vietnam. The mixed methods approach allowed for extension of the quantitative results by the qualitative data [15]. The quantitative survey was used to first assess professionals’ perceptions of the concept of public mental health literacy, and barriers to the mental health literacy development at both the public and individual levels. The focus groups and qualitative data were used to document and describe the specific challenges related to mental health literacy information seeking at the public health level, their possible causes, and suggested solutions to promote mental health literacy at the public health level. The focus groups provided participants the opportunity to provide open commentary and express additional opinions and views on the topic. The study was approved by the U.S. FWA IRB (#18223) at Vietnam National University.

### Participants and recruitment

The goal of the sampling frame was to identify mental health and education professionals with interest and understanding of mental health literacy and the challenges of mental health literacy development at public health level. That is, the goal of the sampling frame was not to obtain a random sample of the general population nor a random sample of mental health professionals, but rather to obtain a sample of mental health professionals actively involved in the professional community and interested in mental health literacy, thus most likely to have an understanding of mental health literacy in Vietnam. Consequently, the study design was structured to recruit participants at the 5^th^ International Conference on Child Mental Health in Hanoi, Vietnam, in October 2019. During one section of the meeting, participants were invited to participate in a session on mental health literacy or a session on developmental disabilities, allowing participants to self-select for interest and background in mental health literacy. Participants selecting the former session were introduced to the study and those interested reviewed and signed the consent form. Participants could decide to take part in either or both sections (quantitative, qualitative) of the study. Of the 85 Vietnamese professionals participating in this portion of the conference, 82 (88% female, mean age = 34 years) completed the quantitative survey; 39% were practicing psychologists, 23% mental health researchers, 17% school counselors, and 11% medical staff (MD or nurses), with a median of nine years working in their field. Forty-eight of these individuals choose to participate in the focus groups, which lasted slightly more than one hour.

### Quantitative survey

The survey assessed basic demographic information, with the primary questions covering four areas, assessing participants’ perceptions regarding the current status in Vietnam of the: (a) availability of effective mental health services; (b) public’s level of mental health literacy, and the difficulty of increasing mental health literacy at the public health vs. individual levels; (c) barriers for the general public finding accurate information about mental health; and the (d) feasibility and effectiveness of different solutions at the individual and public health levels to improve the accuracy and utility of public mental health information [16] (see S1 Appendix for an English version of the measures). Survey items were rated on 3-point or 5-point Likert scale.

### Qualitative data collection procedures

The objective of the focus groups was to provide a more detailed report regarding professionals’ perceptions assessed in the quantitative survey. The 48 Vietnamese professionals were divided into four focus groups that met simultaneously. Each group had one research assistant serving as group moderator, and a second research assistant who audio-recorded the group discussion and took written notes from which the qualitative data were coded. The focus groups took place after a short (15 minutes) introduction to the concept of mental health literacy at the public health level. The focus group moderator’s guide contained four questions, all focused on Vietnam. The focus group questions focused on the public health level, given the value but particular challenges of mental health literacy development at that level: (a) how serious of a challenge development of mental health literacy is at the public health level; (b) what kinds of challenges focus group participants had encountered in their professional work related to providing mental health-related services; (c) what kinds of challenges focus group participants had experienced trying to support development of mental health literacy at the public health level; and (d) how Vietnam can best address these challenges, to increase access to evidence-based mental health treatments (see S2 Appendix for an English version of the focus group questions).

### Data analysis

Survey data were analyzed using SAS 9.4, with descriptive analyses summarizing the data. Focus groups’ audio-recordings were transcribed, then imported into QDA Miner for coding and analysis. Transcripts were coded for themes relating to participants’ opinions and perceptions, including emergent themes and the results were analyzed using QDA Miner. Three researchers independently provided the theme coding, with any discrepancies resolved by group discussion and consensus. All participant comments from the focus groups were translated from Vietnamese into English for this report.

## Results

### I. Vietnamese professionals’ perception of treatment availability

In the survey section focusing on public availability of effective mental health services, 44% of professionals indicated that effective psychotherapy services were minimally or not available in Vietnam, with 75% of professionals indicating that it was difficult or very difficult for lay people to find the effective mental health programs that do exist in Vietnam. In addition, 52% of the professionals indicated that even with their help (i.e., the professional’s help), it was difficult or very difficult for lay people to find effective mental health programs in Vietnam. The qualitative data provided a more in-depth picture, with QDA Miner identifying one theme in this area, a scarcity of such effective services in Vietnam in conjunction with difficulty in finding the few available quality services. One focus group participant commented, for instance “*Psychotherapy services are new and scarce in Vietnam*, *and there is no professional registration of psychologists*. *Even as medical staff*, *I do not know how to find these services outside of the psychiatric hospitals*. *Moreover*, *not all psychiatric hospitals provide psychotherapy*, *only the national hospitals or hospitals in the major cities or provinces”*.

### II. Barriers/challenges in the provision of mental health related services

Focus groups participants were asked about the mental health literacy-related challenges that they encounter in their mental health-related work. Four domains of challenges were raised by the participants (see Table 1). A high frequency of misleading and/or inaccurate information was the top challenge reported, with four sub-themes. The first sub-theme focused on inaccurate information about mental health problems (e.g. their symptoms, causes) in the official mass media in Vietnam (in Vietnam, all TV, radio, and newspaper are governmentally-owned, so there are no “unofficial” mass media). Comments for this sub-theme included “*The national television channel VTV (Vietnam Television) has provided inaccurate information about autism*, *that makes the public think that every mental health problem is autism”; “On the television*, *journalists have said depression and stress cause schizophrenia*”. The second subtheme was related to the stigma-inducing effects of this misleading information. One participant commented *“A newspaper article reported that a man was attacked and killed ‘by a person with mental illness’*, *without saying what the ‘mental illness’ was*. *This kind of report creates misunderstandings about mental health problems*, *that mental illness in general is connected to violence and danger”*. Another participant commented that *“*“*VTV interviewed people about mental health*. *These people were celebrities*, *not experts in mental health*. *The information they communicated was incorrect and highly stigmatizing*. *For example*, *a famous singer said on TV that children will develop ‘mental’ problems such as depression or autism if the children watch too much TV”*. A third subtheme about misleading information in mass media was in regards to conflicting information provided by different media sources, making it difficult for the public to know the actual state of affairs. For instance, one participant said that “*Two newspapers reported on the same child mental health conference*. *One newspaper stated that 20% of Vietnamese children in general had experienced mental illness (which was an accurate summary of the findings)*, *whereas a different newspaper indicated that 20% of Vietnamese students in schools had mental illness*, *implying that academic pressure*, *etc*. *was at least in part responsible for this relatively high rate of mental health problems*. *So it can hard for people to develop an accurate understanding*” The final sub-theme involved misleading mental health postings with a hidden commercial purpose (e.g., on business websites). One participant commented, for instance “*Businesses make money from mental illness by posting misleading information on their websites*, *related to their products or services”*.

**Table 1:** Focus group themes.

Theme raised by focus group	Content of Theme	Occurrences/ Cases^1^
I. Status of effective mental health services in Vietnam	1. Scarcity of and difficulty in finding effective mental health services	3/3
II. Barriers/ challenges to providing mental health services	Misinformation:	8/4
	1. Inaccurate information in official mass media	
	2. Stigma-inducing misinformation	
	3. Conflicting and confusing information in mass media	
	4. Misleading information with hidden commercial purposes	
	5. Belief in non-evidence-based treatments (e.g., traditional healers) and consequent lack of trust in psychotherapy	4/3
	6. Cultural beliefs against talking about personal problems	3/2
	7. Lack of inter-disciplinary collaboration	1/1
III. Status of public Mental Health Literacy (MHL)	Stigma:	17/3
	1. within the Vietnamese word for “mental health”	
	2. towards others	
	3. towards the self	
	4. Lack of MHL among Vietnamese public and leadership	9/3
	5. Minimal concern regarding mental health in society	2/2
IV. Development of MHL at public health level	1. Is difficult	3/3
	2. but is necessary	3/3
V. Barriers / challenges to MHL development at public health level	1. Lack of interest and MHL of upper-level administration and leadership	4/3
	2. Lack of formal mental health policy	3/3
	3. Lack of governmental control over sources of mental health-related information	3/2
	4. Lack of human resources in mental health	3/2
	5. Lack of financial resources	2/2
	6. Lack of collaboration with media	1/1
VI. Potential Solutions	1. Policy advocacy for mental health	9/4
	2. Collaboration with media	7/4
	3. Necessity of focusing on development of a *range* of mental health programs: community programs, mental-health related program (life skills, soft skills, etc.), school-based programs	7/3
	4. Formal information sources on mental health organized by governmental or professional agencies	7/2
	5. Networking with professionals from different sectors (e.g., social enterprise, NGO)	7/3
	6. Development of datasets on mental health services and service coordination	4/3
	7. Raise MHL of leaders	3/3
	8. Psychologists need to advertise their services, in a responsible manner	2/1
	9. Focus specifically on scale up of Vietnamese Evidence Based Treatments	1/1

Notes: *Occurrence* = number of times theme was mentioned during focus groups.

*Case* = number (of four) of focus groups in which sub-theme was mentioned.

The two other major challenges participants raised were (a) the public’s lack of trust and hence valuing of evidence-based mental health treatments, and (b) Vietnamese culture’s valuing not talking about personal problems involving emotions or relationships. The former challenge was reflected in comments such as *“People have no trust in scientific psychotherapy or psychologists*. *If they have problems*, *they will look for help from traditional healers*, *fortune tellers*, *or untrained religious providers*, *or anyone who is known to their family or friends as a “good advisor”*. Other comments in this area focused on the public’s preference for specific non-evidence based treatments, with a participant stating “*Many parents of children with autism trust hyperbaric oxygen treatment*, *or things such as ‘field trip therapy’ provided by special education teachers*. *Given their preference for such treatments with wildly exaggerated effectiveness claims*, *it is very difficult to help parents develop understanding and trust in evidence-based treatment such as ABA with more reasonable claims*”.

In regards to the latter challenge, one participant commented that “*Vietnamese culture says you do not talk about your personal problems*, *your inner-self*, *with anyone*, *which makes coping with and treating these problems quite difficult”*. Another participant stated “*There are Vietnamese proverbs*, *such as ‘if there is a problem*, *the family should close the door and teach each other’*, *or ‘Do not wash your dirty linen in public*’. *Beliefs such as this make people not want to seek professional help for their problems even when it is necessary*”.

### III. Professional’s perceptions of mental health literacy

More than three-quarters (77%) of the professionals in the survey reported that the level of mental health literacy among Vietnamese was low or very low, with a mean rating of 2.2 (SD = 0.48) on a 1 (very low) to 5 (very high) Likert scale. Themes raised within the focus groups on this topic centered around the Vietnamese public’s lack of knowledge, understanding and concern, and high stigma towards mental health problems (Table 1). Specific comments included *“Many Vietnamese people do not even know what mental health is*, *how important it is*, *how it affects our lives*, *even staff and educators at schools don’t know”;* “*I work in the hospital and most of the medical doctors do not have good understanding or knowledge of mental health*”; “*Vietnamese people are unconcerned about mental health*. *They only care about academic achievement or being well behaved in public*”. These issues were seen as particularly relevant in rural areas, exemplified with comments such as “*Vietnamese people in general have limited or no understanding about mental health*, *but this is particularly true in rural areas”*. Three sub-themes related to mental health stigma were identified in the qualitative analyses. The first involved the fact that the formal Vietnamese word for “mental health” (sức khỏe tâm thần) itself is a stigmatized term, somewhat akin to “crazy” or “insane” in English. Participants’ comments in this area included “*The Vietnamese term for ‘mental health’ is primarily associated only with the most severe psychiatric disorders such as schizophrenia*, *and carries with it a negative connotation close to ‘madness’ or ‘crazy’ in English”*; or *“The public has a negative stereotyped attitude towards anything related to the word ‘mental health’*, *even intervention programs*, *or TV or talk shows having the word ‘mental health’ in their title*” The second sub-theme involved public stigma towards individuals with mental health problems. Comments included “*Mental illness is generally seen as an individual’s own fault*, *due to personal weakness*, *or life style choices*. *So individuals with mental health problems experience significant discrimination”; “The Vietnamese public are over-anxious about and over-reactive towards people with mental health problems; for instance*, *if a student says she is depressed*, *her school will isolate her and require a forensic psychiatric evaluation”* (to determine whether it is safe for other students to be around her). The third area identified was self-stigma, with comments such as “*If someone experiences mental health problems*, *they feel guilty and ashamed about their illness*, *and do not want to talk about their experience with their family*, *relatives*, *or anyone*”.

### IV. Professional’s perceptions of the difficulty increasing mental health literacy at the public health vs. individual level

In the next survey question, participants were asked about the relative difficulty of improving mental health literacy at the individual vs. public health levels in Vietnam, rated on a scale of 1 (public health level is much more difficult) to 5 (individual level is much more difficult). Participants were equally split across the scale, with a mean of 3.02 (SD = 1.08), and a skewness of 0.01 indicating a highly symmetric distribution around the midpoint of 3. That is, equal numbers of participants saw the public health level and the individual level as more difficult, with one quarter (26%) indicating that they were equally difficult. However, in the focus groups where the issues were discussed (rather than simply rated as in the survey), participants focused more on difficulties at the public health level, such as effects of a lack of interest, policy, and funding on mental health literacy development: *“Mental health literacy at the public level is difficult*, *because people and even agencies simply do not care about mental health*, *and are not interested in investing in public mental health campaigns”; “Public mental health campaigns are difficult because the Vietnamese government does not have supporting policy*, *so we cannot do campaigns due to a lack of money and human resources*. *In foreign countries such as Australia*, *there have been effective mental health literacy campaigns funded by the government and implemented by governmental agencies”*. The focus groups did, however, identify and emphasize the necessity and advantages of the public health level and challenges associated with the individual level, commenting “*Conducting mental health literacy campaigns at the public health level is difficult but necessary*, *because they can reach large numbers of people”* and “*If we do want to improve the general level of mental health literacy for Vietnamese people*, *working with each individual takes so much time and will not influence many people*”.

### V. Barriers to increasing mental health literacy at the public health level

The quantitative survey assessed professionals’ evaluation of nine different possible barriers (see Table 2) to mental health literacy development at the public health-level. All nine factors were seen as significant barriers by at least 2/3 of the professionals. The three barriers most frequently rated as significant by the professionals were (a) misleading information in the mass media regarding what mental health problems are and their symptoms (83% endorsement as a significant barrier); (b) lack of licensure laws (77%), and (c) the general public’s lack of understanding of what mental health is (leading to a lack of interest in finding valid information) (76%).

**Table 2:** Participant ratings of barriers to the general public finding and accessing effective mental health treatments in Vietnam.

Barrier	Major barrier	Mean^1^ (SD)
1. Misleading information…		
a. in the mass media about mental health problems, and what their symptoms are	83%	4.24(0.67)
b. in the mass media about psychotherapy treatments	71%	4.05(0.88)
c. about how to determine what an effective / helpful treatment is	71%	4.10(0.78)
d. on websites about psychotherapy treatments	67%	4.08(0.85)
5. Lack of licensure laws for non-medical mental health providers such as clinical psychologists, clinical social workers, etc.	77%	4.21(0.87)
6. Lay people’s lack of understanding of causes of mental health problems	76%	4.17(0.81)
7. Lack of effective psychotherapy treatments in Vietnam	75%	4.10(0.90)
8. Stigma preventing people from seeking information about what psychotherapy treatments are helpful/effective.	73%	4.15(0.82)
9. Lack of governmental regulations controlling psychotherapy treatments	70%	4.06(0.91)

Notes

^1^ = On a 1 = *Not a barrier* to 5 = *Major barrier* scale.

In the focus groups, the primary barriers to increasing mental health literacy at the public health level that were identified were: (a) a lack of interest and mental health literacy among upper-level health and education administration and leadership, (b) a lack of mental health policy at local, provincial, and national levels, (c) a lack of governmental control over public sources of mental health-related information, (d) a lack of human resources in mental health, (e) scarcity of financial resources for mental health and (f) limited collaboration with media. One participant stated “*Vietnam Television (the national TV channels) produces mental health reports or shows without collaboration with any mental health professionals*, *making it difficult to raise people’s awareness on this issue*. *Moreover*, *whether the mass media even publishes reports about mental health depends on the personal interest of the organization leaders*.” Another participant commented on the lack of mental health policy and financial resources at the national Ministry of Health, stating “*There is no money for public health campaigns on mental health*. *Mental health simply is not a priority for the Vietnamese government and Ministry of Health*. *The Ministry of Health does not understand the importance of mental health*, *and cares only about physical health”*. In regards to the lack of control over mental health-related information sources, one participant stated “*If you Google mental health in Vietnamese*, *you will find many websites posting articles on mental health*, *most of which are without any scientific validity*. *The quality and sources of the information is totally uncontrolled*”. Another comment within this theme was “*Information about mental health treatment is not controlled or checked regarding its quality or sources*. *Anyone can post information about mental health*, *even highly inaccurate or misleading information*. *Anyone without training or a degree in psychology can claim online or anywhere that they are psychotherapist*, *counselor*, *or psychologist*, *and even provide treatment for clients*”. Finally, regarding the lack of human resources as a barrier to public mental health literacy development, one participant commented, “*Human resources in mental health are very limited*. *Few mental health professionals are competent*, *and most of the qualified professionals only focus on their clinical work*, *they’re not interested in public health work*”.

### VI. Possible solutions

Table 3 lists participants’ quantitative ratings of the feasibility and effectiveness of six possible solutions to improve the accuracy and utility of public mental health information. The majority of participants found all six of the solutions feasible (68% to 91% across the six solutions) and likely to be effective (67% to 78%). The top three solutions in regards to feasibility and likely effectiveness were (a) “conducting public mental health campaigns” (91% rated as feasible or very feasible; 75% rated as likely or very likely effective); (2) “government-approved websites providing accurate information” (90%; 78%, respectively); and (3) “non-profit NGO providing accurate information” (90%; 78%, respectively).

**Table 3:** Solution feasibility and effectiveness.

Solution	Feasibility^1^		Effectiveness^1^	
	Feasible / Very Feasible	Mean(SD)^1^	Effective / Very Effective	Mean(SD)^1^
1. Public health MHL campaigns	91%	1.43 (0.50)	76%	1.24 (0.58)
2. Government-approved websites provide information	90%	1.37 (0.54)	78%	1.22 (0.57)
3. Non-profit NGOs provide accurate information	90%	1.41 (0.55)	78%	1.25 (0.53)
4. Governmental agencies provide accurate information	85%	1.30 (0.61)	74%	1.19 (0.58)
5. Government conflict of interest regulations for advertisements	72%	1.11 (0.71)	73%	1.13 (0.62)
6. Government control of misleading/inaccurate information	68%	0.91 (0.69)	67%	0.95 (0.70)

Notes

^1^On a: 0 = *not feasible/effective*, 1 = *feasible/effective*, 2 = *very feasible/effective* scale.

In the focus groups, participants were invited to suggest public health solutions for increasing mental health literacy (Table 1). The major themes identified in their responses were: (a) policy advocacy for mental health; (b) collaboration with media; (c) the necessity of development of a wide variety of different effective mental health programs; (d) having formal information sources controlled by governmental or governmentally-authorized agencies provide mental health information; (e) professional networking across different sectors beyond health and education (e.g., social enterprises); (f) development of Vietnam database(s) focused on effective mental health services and service coordination; (g) increasing the mental health literacy of political, medical, educational, and media leaders. Within (a), “policy advocacy for mental health”, two sub-themes were identified: (a1) the importance and value of license regulation, and (a2) the importance of explicit and intentional promotion of mental health. In regards to the former, one participant suggested “*We must advocate for mental health policies*, *so that the profession of psychologists is recognized legally*, *and service quality controlled and assured*. *Then people will seek mental health information and support from licensed psychologists and authorized agencies”*. In regards to the latter sub-theme of explicit and intentional promotion of mental health, one participant stated *“Explicit mental health policies are very important*. *Only when there is clear*, *intentional policy from the government will the Ministry of Health and other ministries work together to promote mental health literacy through the mass media or other methods”*.

Comments within the second theme (b) “collaboration with media” focused on different approaches for effective collaboration. One participant suggested “*when we develop mental health campaigns*, *we should collaborate with journalists and other media experts*, *and key opinion leaders*. *The methods of communication should be interesting and varied*, *with interesting photos and videos*. *We could for example invite celebrities to share their own experiences with mental health and mental health challenges*. *We could collaborate and guide them so they would know what was important in regards mental health*, *what they should focus on”*. Another participant suggested *“we could collaborate with radio*, *and do soap operas of stories about kids going through puberty and adolescence to show how normative challenges relate to mental health*. *People could also send in questions or suggestions for the next soap opera episode*. *And we could use a podcast approach as well*. *This could impact on public mental health literacy”*.

Regarding the third theme (c) “development of effective mental health programs”, three sub-themes were identified: (c1) the importance of task-shifting and community mental health programs; (c2) the value mental health-related programs, and (c3) school-based mental health programs. In regards to the first sub-theme, participants suggested “*a program to train traditional healers in basic mental health literacy*, *assessment and intervention could be very useful”*; and “*district health stations could do community education programs for the neighborhood on different mental health topics*, *for example*, *one on depression*, *one on stress*”. The second sub-theme focused on programs that were related to mental health but were not necessarily formal mental health programs, such as “*life skill programs for children”*, *“emotional regulation programs”*, *“how to balance your personal life and job*”, and “*anger management programs for children and young adults*”. In regards to the third sub-theme, school-based programs, one participant suggested “*We should start with mental health psycho-education programs for everyone in the schools*: *administrators*, *staff*, *teachers*, *students*, *parents*, *everyone*”.

Regarding the fourth theme (d) “Information on mental health should be organized by governmental or authorized agencies”, participants suggested *“Governmental agencies such as the National Institute of Mental Health and psychiatric hospitals and NGO in Vietnam such as WHO or UNICEF should have brochures and websites providing accurate information on mental health that could be trusted”;* and *“People trust governmental organizations and universities more than random websites”*. Regarding the fifth theme (e) “Networking across professionals from different sectors, including beyond health and education (e.g., social enterprises)”, one participant suggested *“In order to effectively promote public mental health literacy*, *we must network across mental health professionals*, *educators*, *journalists and media experts*, *even social enterprise agencies that make a profit but share some back to society*”. Comments regarding the sixth theme (f) “Development of Vietnam database(s) on effective mental health services and service coordination” included “*There should be a general public dataset with scientifically-based information regarding mental health problems and treatment*, *and listing of psychologists*, *psychiatrists and other providers*. *This database should be easily accessible and organized by geographical area*, *mental health problem type*, *etc*.*”;* “*We need to develop information systems for mental health professionals and institutions*, *covering providers*, *hospitals*, *social organizations*, *and other relevant areas*, *to increase cross-discipline collaboration”*. Finally, in regards to the seventh (g) theme, “Increasing the mental health literacy of political, medical, educational, and media leaders”, participants stated: *“The solution for public mental health literacy development is to first increase the mental health literacy of the leaders*. *For example*, *school principals*, *directors of provincial Departments of Education*, *or media agencies should be helped to become more aware of the importance of mental health*. *They then will be likely to support implementation of mental health programs in their organizations”; “Even in general hospitals* (i.e., non-psychiatric; emphasis added), *leaders need a good understanding of mental health*, *so that they can facilitate mental health-related services*. *At psychiatric hospitals*, *we need to work with hospital directors to increase their understanding of the importance of public mental health literacy*, *and collaboration on community programs and campaigns*. *At present*, *most psychiatric hospitals focus only on direct within-site services*”.

## Discussion

The first question upon this study focused was Vietnamese mental health and education professionals’ perceptions regarding the current status of mental health services and mental health literacy in Vietnam. There have been a number of similar studies investigating these topics focused on LMIC, including Vietnam [17–19]. The majority of this research, however, was conducted over a decade ago. Given the potential for mental health literacy to change rapidly, as knowledge about mental health changes and as the public’s information exposure and methods of access develop, it is important for data in these areas to be current. Equally important, there has been relatively little research assessing perceptions of professionals regarding these issues. Such information is essential for mental health literacy development at the public health level, as professionals will be the drivers of change in this area, by developing public health programs, advocating for and being implementers of effective policy, etc.

A number of our findings are consistent with previous reports regarding mental health infrastructure in Vietnam. Niemi, Thanh [18], for instance, reported in 2010 an almost complete lack of non-medication (i.e., psychotherapy) mental health treatment options in Vietnam; what little psychotherapy services that existed were not readily available to the general public for a variety of reasons (e.g., they were not covered by national health insurance). This lack of availability may have been due largely to unclear mental health policy at the national level, and an almost complete lack of human resources and funding at all levels [20]. The comments of professionals in our study suggest that the situation has not greatly improved over the past decade. Similarly, the low mental health literacy and high mental health stigma in Vietnam reported by the participants in the present study is consistent with other reports, suggesting that there has been relatively little change in these areas over the past decade [21–23].

Other of our findings, however, are new. Perhaps most importantly, concerns about a lack of accuracy and quality of publicly-available mental health information in Vietnam were a central concern raised by the professionals in our study. They reported, for instance, that a significant portion of the information in the popular media regarding mental health is misleading, including (a) what mental health problems and their symptoms are (e.g., that some media reports suggest that all “mental health” problems are fundamentally autism; that depression always includes delusions); (b) conflicting mental health information across different sources; (c) deceptive information, with a lack of acknowledgment of conflict-of-interest issues (e.g., for-profit agencies’ reports of services provided); and (d) several other issues, as discussed in the Results section. The professionals saw these problems as key factors underlying low public mental health literacy and high public mental health stigma, with the low mental health literacy of political leaders linked to a lack of necessary governmental support for mental health. These perceptions and their implications highlight why assessment of professionals’ perceptions is critical for mental health literacy development at the public health level: Given their knowledge, understanding, and expertise, they can identify fundamental causes (e.g., misinformation in the media) underlying low mental health literacy among the general public, and suggest solutions (as below) for these challenges.

In previous decades, research in HIC has found that the media (as opposed to personal experience) is one of the, if not the, most important sources of information for the public regarding mental health and mental illness [24, 25]. Prior to the dominance of the Internet within the media, Rose [26] found that television was the most powerful medium for framing public consciousness regarding mental health, and that media representations of mental illness could even override people’s own personal experiences regarding how they viewed mental illness. More recently, Peek, Richards [27] found that the Internet is a major source of health information for the public, and that the media has a critical role to play in development of mental health literacy in LMIC [28]; unfortunately, the above concerns raised by the professionals in our study suggest that the positive potential of the media has not yet begun in Vietnam. One underlying cause for this issue identified by the professionals was a lack of collaboration between mental health and media professionals, and increased partnerships with the media were suggested as one approach to increasing public mental health literacy. A related suggested solution was governmental or related agency oversight of mental health information publicly disseminated.

Related to the issue of misinformation in the media, the professionals recommended public as well as professional information systems be developed and enhanced. These suggestions included: (a) a lay public-oriented online database, similar to the Cochrane database, with scientifically-based mental health information but also with a listing of providers evaluated regarding the extent to which they follow a scientific approach; and (b) a parallel database for mental health professionals and institutions regarding current EBT status for mental health assessment and treatment approaches, to enable informed decision-making within the mental health system. This latter recommendation parallels that of the WHO in its *Mental Health Policy and Service Guidance Package* [29] and *Mental Health Global Action Plan for 2013–2020* [30] recommendations. However, these recommendations have not yet been implemented in Vietnam [31].

Another critical barrier identified by the professionals to public access to EBT treatments was a lack of governmental licensure of non-medical mental health professionals, including psychologists. Vietnam is one of the few countries in Asia that does not license or regulate the practice of psychologists and similar mental health professionals [24, 32, 33]. This has several consequences. First, it results in low public confidence in psychological services and few people seek needed psychotherapy services. Second, in most schools and psychiatric hospitals across Vietnam, psychological services are not available and in those where they are, they generally are not valued or respected. Hospital psychologists often only conduct the most basic assessments (e.g., self-report psychopathology questionnaires) that are not considered in the medical decision process. Finally, lack of licensure results in a lack of quality control, in that there is no testing of individuals who will be providing non-medical services (e.g., psychological interventions) for mental health problems, and no ongoing ethical monitoring which helps to maintain basic professionalism.

Finally, contrary to expectations, participants believed that improving mental health literacy at the individual and at the public health levels in Vietnam both would be challenging, of approximate equal difficulty. Ultimately, mental health literacy development at either level has the same goal: increasing people’s mental health literacy. The two approaches differ in their mechanisms but not in this goal. Together this suggests that a detailed analysis of mechanisms (e.g., identifying the cognitive processes related to why media can have a greater impact than personal experience on people’s attitudes towards mental illness) will be important. Such analysis should help to increase the effectiveness and efficiency of mental health literacy development efforts.

The present study has several limitations that should be considered in interpreting our findings. First, a sample of mental health and education professionals attending one child mental health conference in Vietnam was used, potentially limiting generalizability. However, this sampling has the strength that the participating professionals were specifically interested in mental health literacy in Vietnam, and thus were likely knowledgeable with potential influence in this area. Although generalizability to other LMIC in the region is unclear, the results may at least provide a starting point for consideration of these issues in similar countries. Second, a related issue is that the professionals were attending a conference on child mental health, and thus may have been most knowledgeable about child mental health. It is worth noting in this regard, however, that none of the points raised or focus group comments had any direct connection to child (vs. adult) mental health literacy development. Finally, a third limitation was that the challenges to public mental health literacy development identified (e.g., lack licensure for psychologists, leading to a lack of public confidence in psychology) were the participants’ perceptions, and not confirmed with objective assessments. However, although the assessment did not directly assess the objective reality of the situation, it did assess the perceptions of key stakeholders, who will be central in moving the field forward. Despite these two limitations, this study highlights the importance of professionals’ perceptions and key barriers to the development of public mental health literacy. Given the centrality of such professionals’ involvement in public mental health literacy, understanding the barriers they see is critical.

## Supporting information

S1 Appendix Survey questions. (DOCX)

S2 Appendix Focus group questions. (DOCX)

## References

[1] Jorm AF. Mental health literacy: Public knowledge and beliefs about mental disorders. The British Journal of Psychiatry. 2000;177(5):396–401. 10.1192/bjp.177.5.396 11059991

[2] Kutcher S, Wei Y, Coniglio C. Mental health literacy: past, present, and future. The Canadian Journal of Psychiatry. 2016;61(3):154–8. 10.1177/0706743715616609 27254090 PMC4813415

[3] Jung H, von Sternberg K, Davis K. The impact of mental health literacy, stigma, and social support on attitudes toward mental health help-seeking. International Journal of Mental Health Promotion. 2017;19(5):252–67.

[4] Kutcher S, Wei Y, Gilberds H, Brown A, Ubuguyu O, Njau T, et al The African guide: one year impact and outcomes from the implementation of a school mental health literacy curriculum resource in Tanzania. Journal of Education and Training Studies. 2017;5(4):64–73.

[5] de Gooijer W. Trends in EU health care systems: Springer Science & Business Media; 2007.

[6] Rhodes R, Battin M, Silvers A. Medicine and social justice: essays on the distribution of health care: Oxford University Press; 2012.

[7] HMU Central Board of Medicine. Báo Cáo Tổng Kết 30 Năm Đổi Mới Hệ Thống y Tế Việt Nam—Thành Tựu và Thách Thức. (Summary Report of 30 Years of Reform of Vietnam’s Health System—Achievements and Challenges). Hanoi, Vietnam: Nhà xuất bản Y học; 2015; 2015.

[8] Vietnamese Ministry of Health. Chiến Lược Quốc Gia về Sức Khỏe Tâm Thần Giai Đoạn 2016–2025 (National Strategy for Mental Health—2016–2025). Hanoi, Vietnam: NXB Y Tế; 2015.

[9] UNICEF Vietnam. Mental health and psychosocial wellbeing of children and young people in selected provinces and cities in Viet Nam. Hanoi: UNICEF, 2018.

[10] Nguyen AJ, Dang HM, Bui D, Phoeun B, Weiss B. Evaluation of a school-based mental health literacy program in two Southeast Asian countries. School Mental Health. 2020;Paper accepted. 10.1007/s12310-020-09379-6 PMC9053860 35496672

[11] Kaufman AB, Kaufman JC. Pseudoscience: The conspiracy against science: MIT Press; 2018.

[12] The Harvard TH Chan School of Public Health. Superfoods or Superhype?2018 [cited 2020 June, 1]. Available from: https://www.hsph.harvard.edu/nutritionsource/superfoods/.

[13] Zuk P, Zuk P, Lisiewicz-Jakubaszko J. The anti-vaccine movement in Poland: The socio-cultural conditions of the opposition to vaccination and threats to public health. Vaccine. 2019;37(11):1491–4. 10.1016/j.vaccine.2019.01.073 30755366

[14] Nghị định quy định chi tiết và hướng dẫn biện pháp thi hành một số điều của luật bảo hiểm y tế (Vietnamese degree on implementation of health insuarance regluations), 146/2018/NĐ-CP (2018).

[15] Creswell JW, Clark VLP. Designing and Conducting Mixed Methods Research. Thousand Oaks, CA: Sage Publications; 2007.

[16] Weiss B, Pollack AA. Barriers to global health development: An international quantitative survey. PloS one. 2017;12(10). 10.1371/journal.pone.0184846 28972971 PMC5626426

[17] Petersen I, Lund C, Stein DJ. Optimizing mental health services in low-income and middle-income countries. Current opinion in psychiatry. 2011;24(4):318–23. 10.1097/YCO.0b013e3283477afb 21546837

[18] Niemi M, Thanh HT, Tuan T, Falkenberg T. Mental health priorities in Vietnam: a mixed-methods analysis. BMC health services research. 2010;10(1). 10.1186/1472-6963-10-257 20813036 PMC2942883

[19] Saxena S, Maulik PK. Mental health services in low-and middle-income countries: an overview. Current Opinion in Psychiatry. 2003;16(4):437–42.

[20] Vuong DA, Van Ginneken E, Morris J, Ha ST, Busse R. Mental health in Vietnam: burden of disease and availability of services. Asian journal of psychiatry. 2011;4(1):65–70. 10.1016/j.ajp.2011.01.005 23050918

[21] Dang HM, Weiss B, Lam T, Ho H. Mental health literacy and intervention program adaptation in the internationalization of school psychology for Vietnam. Psychology in the Schools,. 2018;55(8):941–54. 10.1002/pits.22156 PMC8986161 35392602

[22] Thai QCN, Nguyen TH. Mental health literacy: knowledge of depression among undergraduate students in Hanoi, Vietnam. International journal of mental health systems. 2018;12(1). 10.1186/s13033-018-0195-1 PMC5937829 29760770

[23] Van der Ham L, Wright P, Van TV, Doan VD, Broerse JE. Perceptions of mental health and help-seeking behavior in an urban community in Vietnam: an explorative study. Community mental health journal. 2011;47(5):574–82. 10.1007/s10597-011-9393-x 21409418 PMC3185226

[24] Coverdale J, Nairn R, Claasen D. Depictions of mental illness in print media: A prospective national sample. Australian and New Zealand Journal of Psychiatry. 2002;36(5):697–700. 10.1046/j.1440-1614.2002.00998.x 12225457

[25] Sieff E. Media frames of mental illnesses: The potential impact of negative frames. Journal of Mental Health. 2003;12(3):259–69.

[26] Rose D. Television, madness and community care. Journal of Community & Applied Social Psychology. 1998;8(3):213–28.

[27] Peek HS, Richards M, Muir O, Chan SR, Caton M, MacMillan C. Blogging and social media for mental health education and advocacy: a review for psychiatrists. Current psychiatry reports. 2015;17(11). 10.1007/s11920-015-0629-2 26377948

[28] Semrau M, Evans-Lacko S, Koschorke M, Ashenafi L, Thornicroft G. Stigma and discrimination related to mental illness in low-and middle-income countries. Epidemiology and psychiatric sciences. 2015;24(5):382–94. 10.1017/S2045796015000359 25937022 PMC8367357

[29] World Health Organization. Mental health information systems. Geneva: WHO; 2005.

[30] World Health Organization. Mental Health Action Plan 2013–2020. Geneva:: WHO; 2013.

[31] Minas H, Edington C, La N, Kakuma R. Mental health in Vietnam In: Minas H, Lewis M, editors. Mental health in Asia and the Pacific. Boston, MA: Springer; 2017 p. 145–61.

[32] Schalkwyk GJV, D’Amato RC. Providing psychological services and counselling in Pacific Rim countries: Where is school psychology in Asia? School Psychology International. 2013;34(2):123–30.

[33] Rich GJ, Jaafar JL, Barron DE. Psychology in Southeast Asia: Sociocultural, Clinical, and Health Perspectives: Routledge; 2020.

