# Supplementary material for: Mental health literacy at the public health level in low and middle income countries: An exploratory mixed methods study in Vietnam
Source: PLoS One. 2020 Dec 31;15(12):e0244573. doi: 10.1371/journal.pone.0244573 (PMC7774916; doi:10.1371/journal.pone.0244573)
Supplement: S1 Appendix — (DOCX) [file pone.0244573.s001.docx]

**S1 Appendix – Survey Questions**

1. Mental health literacy has been defined as knowledge and beliefs about mental health and mental disorders which aid their recognition, management or prevention; and understanding how to obtain and maintain positive mental health; decreasing stigma related to mental disorders; and, enhancing help-seeking efficacy.

In your opinion, in general how high is the mental health literacy of the Vietnamese people?

| 1-very low | 2- Low | 3- Average | 4- High | 5-Very high |
| --- | --- | --- | --- | --- |

2a. Is it easier to improve people’s mental health literacy if you (a) target specific people (e.g., teachers in a particular school) versus (b) target the general public (e.g., all citizens in a city)?

| 1-much easier if targeting specific people | 2-easier if targeting specific people | 3-about the same | 4-easier if targeting the general public | 5-much easier if targeting the general public |
| --- | --- | --- | --- | --- |

2b. Is it easier to improve people’s mental health literacy if you (a) have direct contact with them (e.g. by providing a training to people in a school) versus (b) have contact through a public health campaign (e.g., TV advertisements)?

| 1-much easier if you have direct contact | 2- easier if you have direct contact | 3-about the same | 4- easier if have contact through a public health campaign | 5-much easier if have contact through a public health campaign |
| --- | --- | --- | --- | --- |

2c. If a mental health professional works directly with people (i.e., meets with them personally), how difficult is it to help them find useful information about psychotherapy treatments for mental health problems?

| 1-Very easy | 2- Easy | 3- Average | 4- Difficult | 5-Very difficult |
| --- | --- | --- | --- | --- |

3a. How available in Vietnam are psychotherapy treatments that are helpful for mental health problems (such as OCD, depression, child behavior problems, etc.)?

| 0-Not at all available | 1-A little available | 2- Somewhat available | 3-Fairly available | 4-Very available |
| --- | --- | --- | --- | --- |

3b. How easy is it in Vietnam for lay people to find mental health programs that are likely to be helpful? For example, how easy would it be for a lay person in Vietnam who was having trouble with OCD to find an effective psychotherapy treatment for this in Vietnam?

| 0-Very difficult | 1-Difficult | 2-A little difficult | 3-Easy | 4-Very easy |
| --- | --- | --- | --- | --- |

One problem that mental health professionals face in many countries, including in Vietnam, is how to help the public find mental health psychotherapy programs that are likely to be helpful, when people want or need mental health support. There are many reasons why the public may have difficulty finding programs that will be helpful. In many developing countries such as Vietnam, there are not yet regulations or licensing for mental health service providers (psychologists, social workers, etc.) or for treatment centers. So anyone can call themselves a psychologist or provide services regardless of the quality of the services. Another reason why it can be difficult to find effective mental health services is there often is a large amount of inaccurate information available on the internet, etc. for the public about the effectiveness of mental health programs.

4a. How much of a barrier or impediment to the lay public in Vietnam finding helpful mental health treatments do you think each of the issues below is?

1 – A major barrier

2 – A significant barrier

3 – Somewhat of a barrier

4 – A little bit of a barrier

5 – Not at all a barrier

|  | **Item** |  |  |  |  |  |
| --- | --- | --- | --- | --- | --- | --- |
| 1 | A lack of useful information about psychotherapy treatments in Vietnam | 1 | 2 | 3 | 4 | 5 |
| 2 | Misleading information about psychotherapy treatments | 1 | 2 | 3 | 4 | 5 |
| 2a | Mislabeling of mental health problems, symptoms (e.g., many mental health problems such as ADHD are incorrectly are labeled Autism, which means treatment is provided for the wrong problem) | 1 | 2 | 3 | 4 | 5 |
| 2b | Misleading information about what are helpful/effective treatments for mental health problems | 1 | 2 | 3 | 4 | 5 |
| 2c | Websites provide inaccurate / misleading information about psychotherapy treatments | 1 | 2 | 3 | 4 | 5 |
| 2d | The media (e.g., newspapers, TV) provides inaccurate information about psychotherapy treatments | 1 | 2 | 3 | 4 | 5 |
| 3 | Lack of licensure laws for clinical psychologists, clinical social workers, counselors etc. who provide mental health treatments | 1 | 2 | 3 | 4 | 5 |
| 4 | Lack of governmental regulations controlling psychotherapy treatments | 1 | 2 | 3 | 4 | 5 |
| 5 | Lay people’s lack of understanding of the causes mental health problems like OCD, depression, child behavior problems | 1 | 2 | 3 | 4 | 5 |
| 6 | Stigma prevents people from seeking information about what psychotherapy treatments are helpful and effective. | 1 | 2 | 3 | 4 | 5 |

4b. How feasible and effective do you think the solutions listed below might be to help people find effective psychotherapy treatments? That is, how likely do you think that each of these solutions would be to help lay people distinguish accurate information from misleading information?

|  | Item | **Feasibility** | | | **Effectiveness** | | |
| --- | --- | --- | --- | --- | --- | --- | --- |
|  |  | 0 = Not feasible | 1= Feasible | 2 = Very feasible | 0 = Not effective | 1 = Effective | 2 = Very effective |
| 1 | Government controlling misleading information or inaccurate statements | 0 | 1 | 2 | 0 | 1 | 2 |
| 2 | Public health MHL campaigns (e.g., a public health advertising campaign briefly discussing causes and treatments for depression) | 0 | 1 | 2 | 0 | 1 | 2 |
| 3 | Government-approved websites provide information | 0 | 1 | 2 | 0 | 1 | 2 |
| 4 | Governmental agencies provide accurate information | 0 | 1 | 2 | 0 | 1 | 2 |
| 5 | Non-profit NGOs provide accurate information | 0 | 1 | 2 | 0 | 1 | 2 |
| 6 | Governmental regulations for conflict of interest in advertisements (e.g., requiring that an herbal product advertisement state at the end that the vitamin is not a medication and cannot cure any disease). | 0 | 1 | 2 | 0 | 1 | 2 |
