# Supplementary material for: Mental health literacy at the public health level in low and middle income countries: An exploratory mixed methods study in Vietnam
Source: PLoS One. 2020 Dec 31;15(12):e0244573. doi: 10.1371/journal.pone.0244573 (PMC7774916; doi:10.1371/journal.pone.0244573)
Supplement: S2 Appendix — (DOCX) [file pone.0244573.s002.docx]

**S2 Appendix – Focus Group Questions**

1. How serious of a challenge is development of mental health literacy at the public health level?
2. What kinds of challenges have you encountered in your professional work providing mental health-related services or related activities?
3. What kinds of challenges have you experienced trying to support development of mental health literacy at the public health level?
4. How can Vietnam best address these challenges, so as to increase the public’s access to evidence-based mental health treatments?
